# Statement of Dr. Barrett

**Published:** 1892-08

**Authors:** W. C. Barrett

**Affiliations:** Buffalo, N. Y.


### Domestic Correspondence.

### STATEMENT OF DR. BARRETT.

Buffalo, N. Y., July 13, 1892.

To the Editor of the International Dental Journal:

In the July number of your valuable journal there is an article which, it seems to me, in the endeavor to establish a wholesome principle, places New York educational affairs in a wrong light, and does an unwarranted injury to an old and honored institution. There are a number of reasons which impel me, very much against my will, to indite an answer to it. Error travels faster than truth, and unless the latter starts early she will scarcely keep in sight.

The criticism is directed against my Alma Mater, a university of almost fifty years' standing, which was founded by Austin Flint, Sr., Frank Hamilton, John C. Dalton, and James P. White, and which has always occupied the same high plane upon which they placed it. The University has for years honored me with a place in its Faculty, and loyalty to her demands that I should not cravenly hold my speech when her good name is assailed, through a misapprehension of the real condition.

For years the University had been desirous of establishing a department of dentistry, that her medical teaching might be complete in all its branches. The lack of room has always acted as a bar to the accomplishment of this; but when the new University buildings were perfected, the architect was instructed to include the necessary space.

At the late commencement, the Council unanimously voted to establish the department, and elected four men to form the governing board, and to organize the school upon the same principles under which the other departments were in operation. To signalize the event, to indicate its good will towards dentistry, and to furnish the profession the highest mark of its appreciation, it conferred the honorary degree of Doctor of Dental Surgery upon two men who have for many years been foremost in the work of dental reform and progress, and concerning whose qualifications there can be no question. One of them has for sixteen years been a member of the Dental Examining Board of the State of New York, and for a number of years its president. The other has for nearly a quarter of a century been in a most reputable practice, a leading dental society man, and the peer of any dentist in the State. As an operator his name stands at the head in western New York.

It is not the fact that the degrees were conferred for the purpose of qualifying them for a position in the Faculty, for both already held the State degree, which, by the peculiar enactments of our Legislature, is the highest qualification that any man can hold in the State of New York. Even this was not necessary to a position in the Faculty. No degree is essential to a professorship in any dental or medical college in any State. The signature on a diploma of a professor who holds no degree whatever is equally good with that of the man who has all that every school can offer.

There is no legislation forbidding the granting of honorary degrees in dentistry. The schools represented in the National Association of Dental Faculties voluntarily agreed between themselves that they would not confer it without the consent of a majority of those represented, but that is far from being an interdiction of the degree. The University of Buffalo owes no allegiance to that body, as yet. When its dental department becomes a member, it will be governed by its rules and regulations, and will sustain them to the best of its ability. In the late instance the University conferred the honorary degrees in perfect good faith, and without the slightest thought of doing anything that would subject itself to criticism.

Nor would the question have been raised in this case any more than it has in the numerous instances that have occurred in the past, had it not been for a misconception of the situation by the local opposition, without which no college ever was established. There is scarcely a school that has not upon its Faculty men who hold honorary degrees, and in some they make up a large proportion of the teaching force. If Buffalo is to be censured for this, there is not a school in existence which will not lie under the same disability, for the late degrees were not conferred in the face of any legislation to the contrary, and the status of its teachers is precisely that of the other institutions. There has never, to my knowledge, been any expression in any competent dental body of any hostility to this form of diploma.

Honorary degrees have been recognized in all ages and by all schools. The English universities grant them, and distinguished clergymen, and other professional men, may be found in all countries, who are proud of the D.C.L. conferred by the old world universities. Eminent teachers feel honored at the reception of the LL.D. Our own universities confer the D.D. upon teachers and others, and the M.D. upon many men not in medical practice. The D.D.S. has perhaps been showered too freely by our young schools, but the degree itself is young, and the position of those who have received it has never yet been questioned.

Was the deceased Edward Maynard, of Washington, held in less repute because all of his numerous degrees were honorary? Was the late James W. White ever called to account because, in recognition of his eminent attainments, the Pennsylvania College of Dental Surgery conferred upon him the honorary degree of D.D.S., and a New York university that of M.A.? Good old Dartmouth has lately given to Dr. R. R. Andrews, of Cambridge, the honorary degree of M.A. Is there a dentist worthy the name who does not feel reflected upon himself a little of the distribution so worthily bestowed? It is an honor to the whole profession, and should be so considered. Will the INTERNATIONAL DENTAL JOURNAL proceed to call that school to account for it? Michigan University conferred the honorary degree of Ph.D. upon W. D. Miller. Does the JOURNAL propose to censure that school for this act, or for granting the degrees of M.D. or D.D.S. to honored dentists? If every eminent man who holds an honorary degree in dentistry is to be cast out therefor, it will be a lonesome time for the comparatively few remaining, for the highest have been singled out for this distinction. Until some legislation by some competent professional body forbids the granting of degrees of honor by the universities, which too often are beyond their jurisdiction, all the honorary diplomas are alike, and there can be no preference shown to any, whether early or late.

Personally, I have been opposed to the practice which has been followed by schools in all ages. So far as I know, I am the only man who has publicly placed himself on record against degrees not in course. But until all the schools accept my views and agree to be governed by my dictation, they are not to be censured for doing the things which I may individually condemn. It is barely possible that there may be nothing criminal in acts which do not meet my personal approval. The colleges, at any rate, are not more responsible for my utterances than I am for their proceedings. I shall do all that I can to bring about the reforms which I deem advisable, but until some united action can be induced I shall recognize the present condition, and shall not proclaim war against everything that now is. I propose to live in this world for a while longer, and if I do so I must conform to what others deem best, and not demand my own way in all things.

Some years since, when editor of the *Independent Practitioner*, I made an attack upon the practice of granting the regular degree of the schools *sine curriculo*, upon a mere examination, and the outcome of the matter was the formation of the National Association of Dental Faculties, and the abandonment of the practice complained of. The question of honorary degrees never entered into the discussion. I contended that it was wrong to confer the regular degree upon one who had not gone through the curriculum of the schools. I am still of that opinion, and I am happy to say that there are very few who would now differ with me. The degrees conferred by the University of Buffalo were not of that character, for they did not pretend to be degrees in course, and were not conferred as such. This form of diploma has not, therefore, been "revived in a neighboring State," for there has been no pretence of conferring a degree in course.

There was no "embarrassing difficulty," because the men were already legally and professionally qualified for the positions which they afterwards assumed. It was simply an effort, in perfect good faith, to exhibit to dentistry a feeling of professional good will and fraternal fellowship, and as such should have been accepted unquestioned, as many such have been before on the part of other old universities and colleges.

I can assure you that it is only that there may be a proper comprehension of the truth in this case, and because the one side having been laid before your readers I deemed it but fair that the other side should be heard also, that I have addressed to you this communication. The school is already established, and has placed its standard as high as that of the highest, and it proposes, so far as I know, to assist, according to its ability, in keeping the reputation of our profession unspotted.

I am very truly yours,

W. C. Barrett.

